# Boson peak, elasticity, and glass transition temperature in polymer glasses: Effects of the rigidity of chain bending

**DOI:** 10.1038/s41598-019-55564-2

**Published:** 2019-12-20

**Authors:** Naoya Tomoshige, Hideyuki Mizuno, Tatsuya Mori, Kang Kim, Nobuyuki Matubayasi

**Affiliations:** 1 Division of Chemical Engineering, Graduate School of Engineering Science, Osaka University, Toyonaka, Osaka 560-8531 Japan; 2 Graduate School of Arts and Sciences, The University of Tokyo, Tokyo, 153-8902 Japan; 3 Division of Materials Science, University of Tsukuba, 1-1-1 Tennodai, Tsukuba, Ibaraki 305-8573 Japan; 4 Elements Strategy Initiative for Catalysts and Batteries, Kyoto University, Katsura, Kyoto 615-8520 Japan

**Keywords:** Glasses, Polymers

## Abstract

The excess low-frequency vibrational spectrum, called boson peak, and non-affine elastic response are the most important particularities of glasses. Herein, the vibrational and mechanical properties of polymeric glasses are examined by using coarse-grained molecular dynamics simulations, with particular attention to the effects of the bending rigidity of the polymer chains. As the rigidity increases, the system undergoes a glass transition at a higher temperature (under a constant pressure), which decreases the density of the glass phase. The elastic moduli, which are controlled by the decrease of the density and the increase of the rigidity, show a non-monotonic dependence on the rigidity of the polymer chain that arises from the non-affine component. Moreover, a clear boson peak is observed in the vibrational density of states, which depends on the macroscopic shear modulus *G*. In particular, the boson peak frequency ω_BP_ is proportional to $\sqrt{G}$. These results provide a positive correlation between the boson peak, shear elasticity, and the glass transition temperature.

## Introduction

Glasses show vibrational and mechanical properties that are markedly different from other crystalline materials^1,2^. Thermal measurements and scattering experiments have been performed to study the properties of various glassy systems, such as covalent-bonding^3–8^, molecular^9–13^, metallic^14–17^, and polymeric^18–23^ glasses. For instance, the excess vibrational modes at low frequencies and the excess heat capacity at low temperatures exceeding the Debye predictions, which describe the corresponding crystalline values, have been observed universally in various glassy materials. This phenomenon, which is referred to as the boson peak (BP), has been widely studied.

The ideas of elastic heterogeneities^24–26^ and criticality near isostatic state and marginally stable state^27–30^ have been introduced, following the recent theoretical advances for understanding the origin of anomalies in glasses. Based on these theories, the mean-field formulations have been developed by using the effective medium technique^24–26,29,30^. In addition, more recent studies^31,32^ have focused on the local inversion-symmetry breaking, which can explain the microscopic origin of the BP. The anomalous vibrational properties in both crystals and glasses have also been investigated within the framework of the phonon Green's function^33^.

Molecular dynamics (MD) simulations play an essential role for studying the vibrational and mechanical properties of glasses. Firstly, MD simulations enable to assess the theoretical predictions. In fact, various MD simulations have been performed on simple atomic glasses, e.g., Lennard-Jones (LJ) systems^34–40^. Concerning the isostaticity and marginal stability^27–30^, the systems with a finite-ranged, purely repulsive potential have also been studied^41–44^, and are considered as the simplest model of glasses. In particular, it is crucial for MD simulations to solve finite-dimensional effects that are not captured by the mean-field treatments^45–47^. Secondly, MD simulations perform quasi-experiments on well-defined systems and access data that cannot be examined experimentally. Relevant systems to experiments and applications have been simulated, including covalent-bonding^48–53^, metallic^54–57^, polymeric^58–62^ glasses. These simulation studies complete theoretical understandings based on simple systems and experimental observations of more complex systems.

The vibrational properties and the BP of polymeric glasses have been studied by both of experiments^18–23^ and MD simulations^58–62^. The effects caused by non-covalent bonds including bending forces and chain length represent an important feature of polymer glasses. Previous experiments^18,19^ have investigated the effects of the pressure or densification on the frequency and intensity of the BP in polymeric glasses. It was demonstrated that the evolution of the BP with pressure cannot be scaled by the Debye values (i.e., the Debye frequency and the Debye level). Therefore, the pressure effects cannot be explained only by the variation of macroscopic elasticity. In contrast, another experiment^20^ has shown that the polymerization effects on the BP is explained by the change in macroscopic elasticity as the frequency and intensity variations of the BP are both scaled by the Debye values.

In addition, Zaccone *et al*. have recently performed MD simulations to calculate the vibrational density of states (vDOS) in polymeric glasses by changing the chain length and the rigidity of the chain bending^61^. This work studied the vibrational eigenstates in a wide range of frequencies and the effects of the chain length and bending rigidity on the high-frequency spectra. Furthermore, Giuntoli and Leporini studied the BP of polymeric glasses having chains with highly rigid bonds^62^. It was demonstrated that the BP decouples with macroscopic elasticity and arises from non-bonding interactions only. Although these studies^61,62^ have helped understand polymeric glass properties, the effects of bending rigidity and chain length on the low-frequency spectra and BP need to be further studied.

Herein, the vDOS and the elastic moduli of polymeric glasses are analyzed through coarse-grained MD simulations (see Methods). In particular, the connection between the BP and elasticity as well as the glass transition temperature is explored by systematically changing the bending stiffness of short and long polymer chains. The contributions of the present study are given as follows. We demonstrate that polymeric glasses can exhibit extremely-large non-affine elastic response (compared to atomic glasses), whereas the BP is simply scaled by the behavior of macroscopic shear modulus. This behavior of the BP can be explained by the theory of elastic heterogeneities^24–26^. Our results indicate that effects of the bending rigidity on the BP are encompassed in change of macroscopic elasticity, which is in contrast to effects of pressure^18,19^, but instead is similar to effects of polymerization^20^. Furthermore, we show the positive correlation among the BP, elasticity, and the glass transition temperature. Finally, we will discuss the relaxation dynamics in the liquid state, in relation to our results of low-frequency vibrational spectra.

## Results

### Glass transition temperature

**Figure 1:**
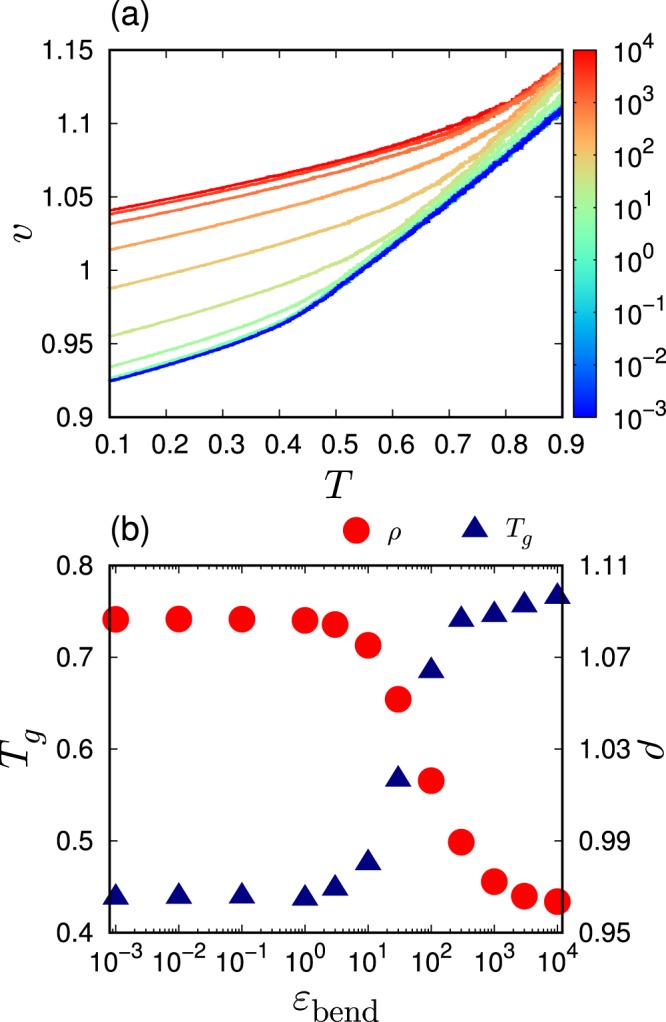
Glass transition temperature and density in the glass state. (**a**) The specific volume $v$ versus the temperature $T$ during the process that the system is cooled down from the liquid state to the glass state. The color of line indicates the value of bending rigidity ${\varepsilon }_{{\rm{bend}}}$ according to the color bar. (**b**) Glass transition temperature ${T}_{g}$ (triangles) and density $\rho$ at zero temperature after the glass transition (circles) are plotted against ${\varepsilon }_{{\rm{bend}}}$. The chain length is $L=50$.

When the polymeric system is cooled down from the liquid state under a constant pressure, the volume of the system monotonically decreases with decreasing the temperature. Figure 1a shows the specific volume $v$ as a function of the temperature $T$ for several different bending rigidities ${\varepsilon }_{{\rm{bend}}}$ and the chain length $L=50$. For each value of ${\varepsilon }_{{\rm{bend}}}$, the slope of the $v$-$T$ curve clearly presents a discontinuous change at a certain temperature, which is defined as the glass transition temperature ${T}_{g}$. Figure 1b (triangles) presents the value of ${T}_{g}$ as a function of ${\varepsilon }_{{\rm{bend}}}$. As the rigidity increases from ${\varepsilon }_{{\rm{bend}}}=1$ to $1{0}^{3}$, ${T}_{g}$ progressively increases from ${T}_{g}\simeq 0.45$ to 0.75. Below $\varepsilon =1$ and above $\varepsilon =1{0}^{3}$, the variation of ${T}_{g}$ is low or even negligible. In addition, Fig. 1b (circles) plots the density $\rho (=1/v)$ of the system that is quenched down to $T=0$. The density decreases from $\rho \simeq 1.09$ to 0.97 as the rigidity increases from ${\varepsilon }_{{\rm{bend}}}=1$ to $1{0}^{3}$. As the chain bending becomes rigid, the glass transition occurs at a higher temperature, and as a result, the density in the glass state becomes lower. The similar observation was obtained by Milkus *et al.*^61^.

**Figure 2:**
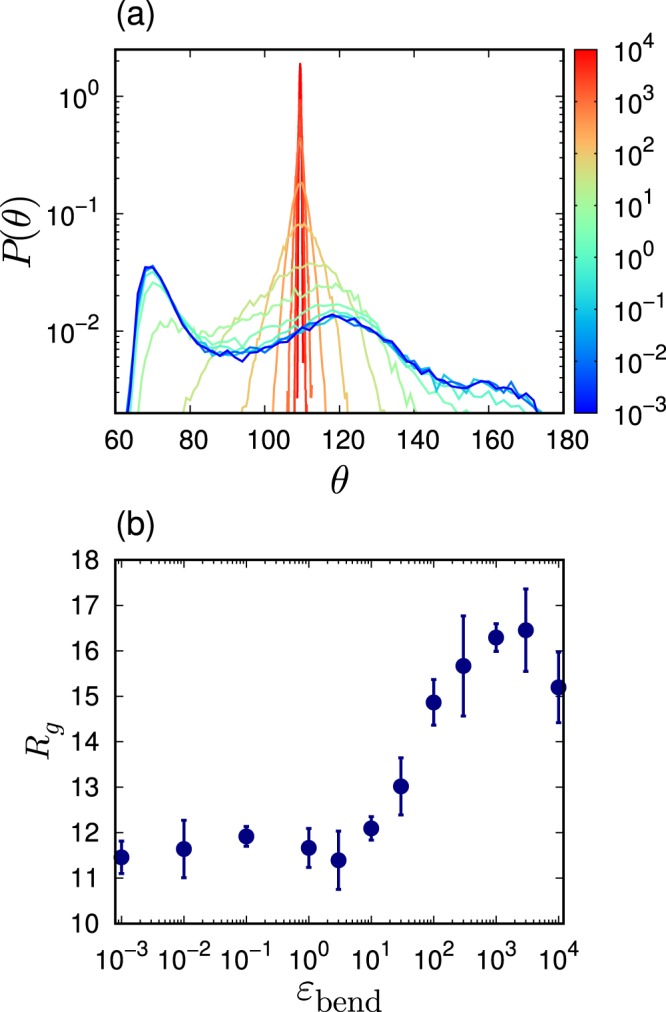
Conformation of polymeric chains. (**a**) Probability distribution of angle formed by three consecutive beads along the chain, $P(\theta )$, is presented for several different rigidities ${\varepsilon }_{{\rm{bend}}}$. The color of line indicates the value of bending rigidity ${\varepsilon }_{{\rm{bend}}}$ according to the color bar. (**b**) Radius of inertia ${R}_{g}$ is plotted as a function of ${\varepsilon }_{{\rm{bend}}}$. The chain length is $L=50$.

These behaviors of ${T}_{g}$ and $\rho$ can be understood by studying the microscopic conformation of the polymeric chains. Figure 2a presents the probability distribution of the angle formed by three consecutive beads along the chain, $P(\theta )$, when changing the rigidity ${\varepsilon }_{{\rm{bend}}}$. Two peaks are observed at approximately $\theta \simeq 7{0}^{\circ }$ and $\theta \simeq 12{0}^{\circ }$ for a low rigidity (${\varepsilon }_{{\rm{bend}}}\le 1$). A similar distribution $P(\theta )$ was also reported in ref. ^61^. As the rigidity increases, the peak position in $P(\theta )$ shifts towards ${\theta }_{0}=109.{5}^{\circ }$. It is noted that the bending potential ${U}_{{\rm{bend}}}(\theta )$ in Eq. 5 tends to stabilize the angle $\theta$ at ${\theta }_{0}=109.{5}^{\circ }$. In addition, Fig. 2b presents the radius of gyration ${R}_{g}$ as a function of ${\varepsilon }_{{\rm{bend}}}$. It can be observed that ${R}_{g}$ increases from ${R}_{g}\simeq 11.5$ to 16.5 with an increasing ${\varepsilon }_{{\rm{bend}}}$. Importantly, these variations of conformation are induced intensively when the rigidity increases from ${\varepsilon }_{{\rm{bend}}}=1$ to $1{0}^{3}$, which exactly matches the region where variations of ${T}_{g}$ and $\rho$ are observed in Fig. 1b. Therefore, it can be concluded that the conformation changes of the polymeric chains control the glass transition temperature and the density. In fact, as the rigidity of the chain bending increases, the angle $\theta$ of the polymer chains tends to be stabilized at ${\theta }_{0}=109.{5}^{\circ }$ and the radius of inertia increases. As a result, the glass transition occurs at a higher temperature and the lower density (larger volume). At ${\varepsilon }_{{\rm{bend}}}\lesssim 1$, the effect of the bending interaction of Eq. 5 is weak compared to those of the LJ and finitely extensible nonlinear elastic (FENE) components of Eqs. 3 and 4 (see Methods). However, at ${\varepsilon }_{{\rm{bend}}}\gtrsim 1{0}^{3}$, the opposite phenomenon occurs.

It is noted that the glass transition occurs at a lower temperature for $L=3$ than for $L=50$, which is consistent with a previous report^64^. Correspondingly, the values of $\rho$ for $L=3$ becomes larger than that of $L=50$. However, common results were observed between $L=3$ and 50 with respect to the dependences on the rigidity ${\varepsilon }_{{\rm{bend}}}$. Specifically, ${T}_{g}$ and $\rho$, as well as the conformation of the polymeric chains progressively change when the rigidity increases from ${\varepsilon }_{{\rm{bend}}}=1$ to $1{0}^{3}$, which also occurs for $L=50$.

### Elastic properties

**Figure 3:**
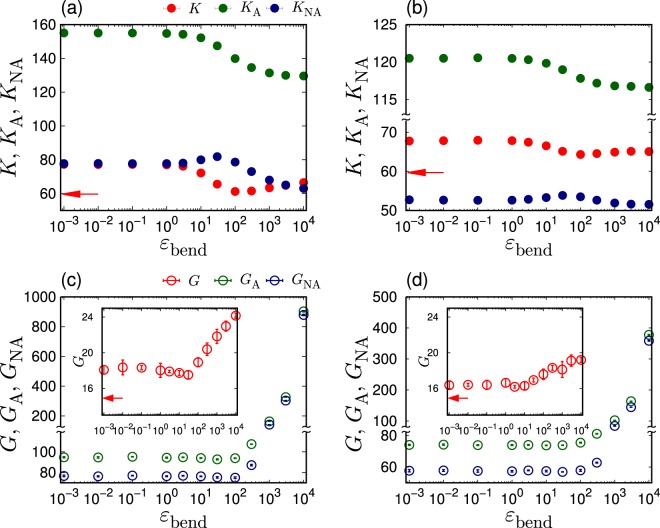
Elastic properties of polymeric glasses. Plots of the bulk modulus $K$ [(**a**,**b**) upper panels] and the shear modulus $G$ [(**c**,**d**) bottom panels] as functions of the strength of bending rigidity ${\varepsilon }_{{\rm{bend}}}$. The chain length is $L=50$ [(**a**),(**c**) left panels] and $L=3$ [(**b**,**d**) right panels]. In the figures, we also plot the affine moduli, ${K}_{{\rm{A}}}$ and ${G}_{{\rm{A}}}$, and the non-affine moduli, ${K}_{{\rm{NA}}}$ and ${G}_{{\rm{NA}}}$. The horizontal arrows indicate the values, $K=59.7$ and $G=14.9$, of atomic LJ glasses that are extracted from ref. ^63^.

The elastic properties of polymer glasses are studied by changing the strength of bending rigidity. An external strain is applied to the system at $T=0$, which enables to measure the corresponding elastic moduli. Specifically, the volume-changing bulk deformation and the volume-conserving shear deformation are applied, which provide the bulk modulus $K$ and the shear modulus $G$, respectively^63^. Figure 3 presents the values of $K$ and $G$ as functions of ${\varepsilon }_{{\rm{bend}}}$. Disordered systems exhibit large non-affine elastic responses^2^. The elastic moduli, $M=K$ and $G$, are decomposed into affine moduli ${M}_{{\rm{A}}}$ and non-affine moduli ${M}_{{\rm{NA}}}$, i.e., $M={M}_{{\rm{A}}}-{M}_{{\rm{NA}}}$^65–67^. In Fig. 3, these affine and non-affine components are also presented.

First, the bulk modulus $K$ is analyzed for $L=50$ and presented Fig. 3a. The affine component ${K}_{{\rm{A}}}$ decreases from ${K}_{{\rm{A}}}\simeq 155$ to 130 as ${\varepsilon }_{{\rm{bend}}}$ changes from 1 to $1{0}^{3}$. The reduction of ${K}_{{\rm{A}}}$ is caused by the decrease of the density $\rho$ with the increasing ${\varepsilon }_{{\rm{bend}}}$ (see Fig. 1b). In contrast, the non-affine component ${K}_{{\rm{NA}}}$ shows a non-monotonic dependence on the ${\varepsilon }_{{\rm{bend}}}$. In particular, ${K}_{{\rm{NA}}}$ slightly increases from ${\varepsilon }_{{\rm{bend}}}=1$ to 30, which is induced by the decrease of the density $\rho$. As ${\varepsilon }_{{\rm{bend}}}$ is further increased above ${\varepsilon }_{{\rm{bend}}}=30$, ${K}_{{\rm{NA}}}$ decreases. This is because the non-affine relaxation process is constrained due to the large rigidity of ${\varepsilon }_{{\rm{bend}}}$. As a result, the total modulus of $K={K}_{{\rm{A}}}-{K}_{{\rm{NA}}}$ also presents a non-monotonic behavior, which is demonstrated in Fig. 3a. From ${\varepsilon }_{{\rm{bend}}}=1$ to $1{0}^{2}$, $K$ decreases from $K\simeq 80$ to 60, which is caused by the reduction of ${K}_{{\rm{A}}}$. Moreover, $K$ increases from $K\simeq 60$ to 65 above ${\varepsilon }_{{\rm{bend}}}=100$, which is caused by the reduction of ${K}_{{\rm{NA}}}$. Therefore, the ${\varepsilon }_{{\rm{bend}}}$ dependence of the bulk modulus $K$ is determined by the competition between the density reduction and the increase in the bending rigidity.

Further, the shear modulus $G$ is analyzed for $L=50$ and presented Fig. 3c. We note that a non-monotonic behavior of the shear modulus as a function of bending stiffness is observed in the previous study^60^. Figure 3c demonstrates that the bending rigidity strongly affects the shear modulus compared to the bulk modulus. Particularly, above ${\varepsilon }_{{\rm{bend}}}=1{0}^{2}$, both of the affine ${G}_{{\rm{A}}}$ and non-affine ${G}_{{\rm{NA}}}$ components considerably increase. As the shear deformation is anisotropic and causes deformations of the angles $\theta$ of polymeric chains, its response is expected to be highly affected by the bending rigidity. Interestingly, contrary to the important increases of ${G}_{{\rm{A}}}$ and ${G}_{{\rm{NA}}}$, the total shear modulus $G={G}_{{\rm{A}}}-{G}_{{\rm{NA}}}$ shows a low variation (by comparing ${G}_{{\rm{A}}}\simeq {G}_{{\rm{NA}}}\simeq 900$ with $G\simeq 24$ at ${\varepsilon }_{{\rm{b}}{\rm{e}}{\rm{n}}{\rm{d}}}=1{0}^{4}$). The bending rigidity increases the affine shear modulus but, at the same time, the non-affine component also increases to cancel the increase in ${G}_{{\rm{A}}}$, and as a result, the total shear modulus presents a low increase. The elasticity of the shear deformation is therefore different from that of the bulk deformation, which is obvious when the elastic moduli are decomposed into affine and non-affine components.

Figure 3 also shows $K$ in (b) and $G$ in (d) for $L=3$. The values of $K$ and $G$ of $L=3$ are smaller than those of $L=50$, due to the bonding energy, ${\varepsilon }_{{\rm{FENE}}}$, connecting the monomers along the polymeric chains. The responses of $K$ and $G$ to the variation of ${\varepsilon }_{{\rm{bend}}}$ are also weaker for $L=3$. However, $K$ and $G$, as well as affine ${K}_{{\rm{A}}}$ and ${G}_{{\rm{A}}}$ and non-affine ${K}_{{\rm{NA}}}$ and ${G}_{{\rm{NA}}}$, exhibit overall common dependences on ${\varepsilon }_{{\rm{bend}}}$ between $L=3$ and 50. Therefore, the decrease in $\rho$ and increase in ${\varepsilon }_{{\rm{bend}}}$ engenders similar effects on the elasticity for $L=3$ and 50.

Finally, it is remarked that the polymer glasses present larger non-affine elastic components than the atomic (LJ) glasses^63,68^. Even under an isotropic bulk deformation, the non-affine ${K}_{{\rm{NA}}}$ ($\simeq 80$ for $L=50$ and $\simeq 50$ for $L=3$, at ${\varepsilon }_{{\rm{bend}}}\le 1$) is approximately half of the magnitude of the affine ${K}_{{\rm{A}}}$ ($\simeq 155$ for $L=50$ and $\simeq 120$ for $L=3$, at ${\varepsilon }_{{\rm{bend}}}\le 1$). This result is different from that of the LJ glasses, where a negligible value of ${K}_{{\rm{NA}}}\simeq 0.5$ (whereas ${K}_{{\rm{A}}}\simeq 60.2$) was obtained^63^. Larger non-affine moduli reflect various elastic responses due to the multiple degrees of conformations in polymeric chains. Therefore, the non-affine deformation process must be considered to characterize the elastic property of polymeric systems. Interestingly, ref. ^69^ has reported that non-affine displacements also play an important role on melting of amorphous polymers.

### Low-frequency vibrational spectra

#### Reduced vDOS

Finally, the spectra of vibrational eigenmodes in polymer glasses are studied. The vibrational mode analysis is performed on the configuration of the polymeric system at $T=0$, which corresponds to the inherent structure^71,72^. The Hessian matrix is diagonalized to obtain the eigenfrequencies ${\omega }^{k}$ that corresponds to the square root of the eigenvalues ${\lambda }^{k}$, i.e., ${\omega }^{k}=\sqrt{{\lambda }^{k}}$ ($k=1,2,...,3{N}_{{\rm{p}}}$). The specific expression of the Hessian matrix is given in Supplementary Material.

**Figure 4:**
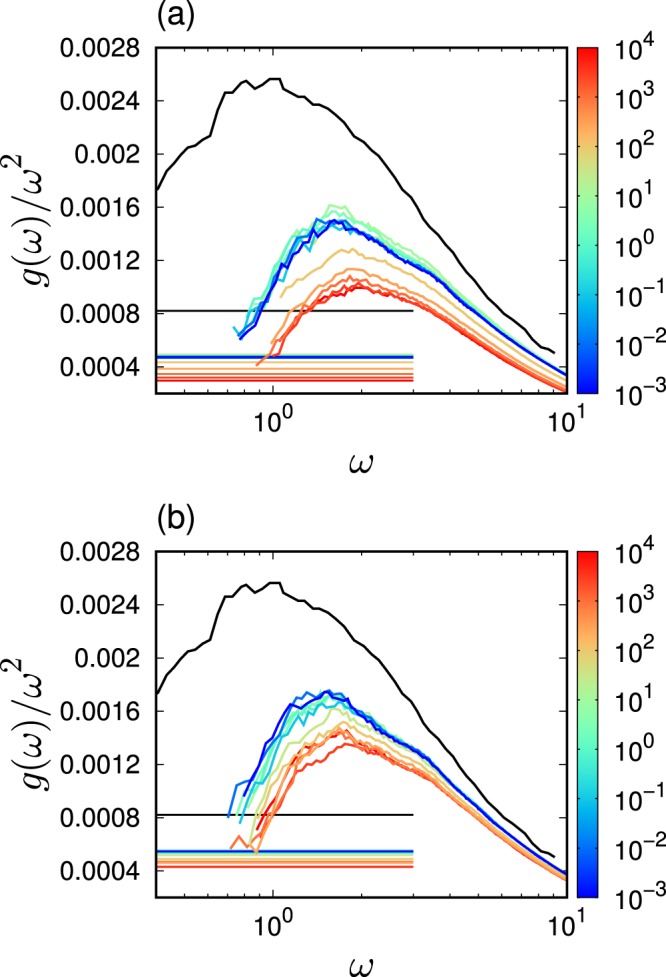
Low-frequency vibrational spectra. We plot the vDOS $g(\omega )$ divided by ${\omega }^{2}$, i.e., the reduced vDOS $g(\omega )/{\omega }^{2}$, with changing the strength of bending rigidity ${\varepsilon }_{{\rm{bend}}}$. The chain length is (**a**) $L=50$ and (**b**) $L=3$. The horizontal lines indicate the Debye level ${A}_{{\rm{D}}}$. The color of line indicates the value of bending rigidity ${\varepsilon }_{{\rm{bend}}}$ according to the color bar. Black lines present value of the LJ glass which is taken from ref. ^70^.

**Figure 5:**
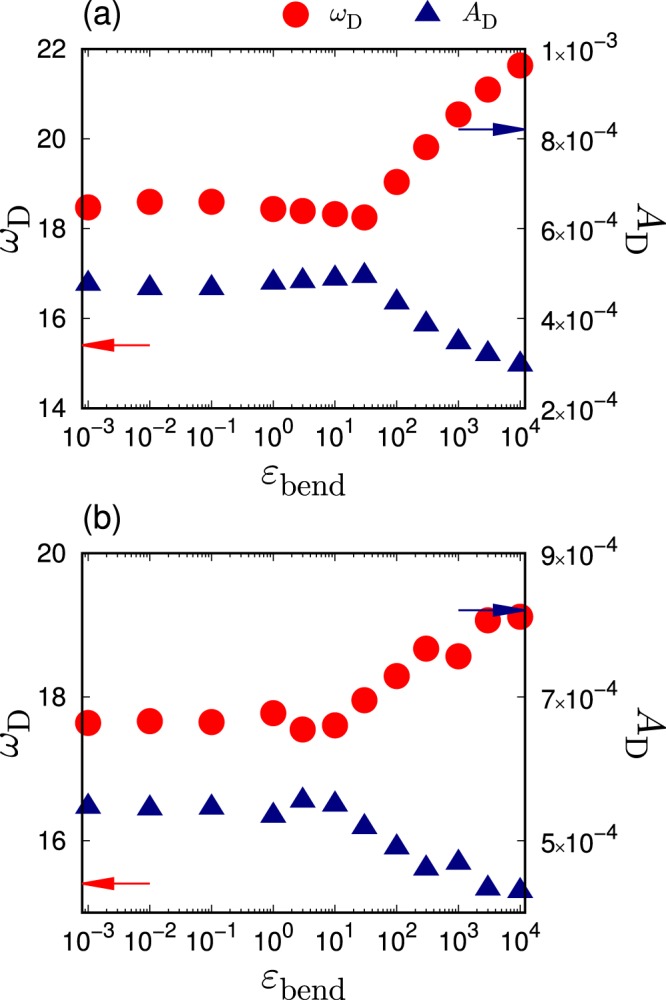
Debye frequency and Debye level. Plots of the Debye frequency ${\omega }_{{\rm{D}}}$ (circles) and the Debye level ${A}_{{\rm{D}}}=3/{\omega }_{{\rm{D}}}^{3}$ (triangles) as functions of the strength of bending rigidity ${\varepsilon }_{{\rm{bend}}}$. The chain length is (**a**) $L=50$ and (**b**) $L=3$. The values of ${\omega }_{{\rm{D}}}$ and ${A}_{{\rm{D}}}$ are calculated from the elastic moduli of $K$ and $G$ that are presented in Fig. 3. The arrows indicate values of atomic LJ glasses that are taken from ref. ^70^.

The statistics of the eigenfrequency provide the vDOS, $g(\omega )$. Figure 4 presents the reduced version of the vDOS, $g(\omega )/{\omega }^{2}$, when changing the rigidity ${\varepsilon }_{{\rm{bend}}}$ and for $L=50$ in (a) and $L=3$ in (b). The reduced vDOS, $g(\omega )/{\omega }^{2}$, of the Debye theory is the so-called Debye level ${A}_{{\rm{D}}}$^71,72^. ${A}_{{\rm{D}}}$ is calculated from the elastic moduli, $K$ and $G$, as follows: ${A}_{{\rm{D}}}=3/{\omega }_{{\rm{D}}}^{3}$, where ${\omega }_{{\rm{D}}}$ is the Debye frequency defined as ${\omega }_{{\rm{D}}}={\left[18{\pi }^{2}\rho /(2{{c}_{{\rm{T}}}}^{-3}+{{c}_{{\rm{L}}}}^{-3})\right]}^{1/3}$, and ${c}_{{\rm{L}}}=\sqrt{(K+4G/3)/\rho }$ and ${c}_{{\rm{T}}}=\sqrt{G/\rho }$ are the longitudinal and transverse sound speeds, respectively. Figure 5 presents the values of ${\omega }_{{\rm{D}}}$ and ${A}_{{\rm{D}}}$ as functions of ${\varepsilon }_{{\rm{bend}}}$. As the bulk modulus is approximately four times larger than the shear modulus, ${\omega }_{{\rm{D}}}$ and ${A}_{{\rm{D}}}$ are mostly determined with the shear modulus, i.e, ${\omega }_{{\rm{D}}}\approx {(9{\pi }^{2}\rho )}^{1/3}{c}_{{\rm{T}}}$ and ${A}_{{\rm{D}}}\approx 1/(3{\pi }^{2}\rho {c}_{{\rm{T}}}^{3})$.

As shown in Fig. 4, the polymer glasses present clear excess peaks over the Debye level, i.e., the BP. The BP frequency, ${\omega }_{{\rm{BP}}}$, is defined as the frequency at which $g(\omega )/{\omega }^{2}$ is maximal. As ${\varepsilon }_{{\rm{bend}}}$ increases, ${\omega }_{{\rm{BP}}}$ shifts to a higher frequency. In addition, the height of the reduced vDOS, $g({\omega }_{{\rm{BP}}})/{\omega }_{{\rm{BP}}}^{2}$, becomes lower. These behaviors of BP are consistent with the previous observation^61^. These shifts are observed in the region from ${\varepsilon }_{{\rm{bend}}}=10$ to $1{0}^{3}$ for $L=50$ and 3. Importantly, this region corresponds to the shear modulus $G$ variations, as shown in Fig. 3c,d. As the bulk modulus is much larger than the shear modulus, the bulk modulus should only have minor effects on the low-frequency spectra. Therefore, the BP of the proposed system should only be controlled by the shear elasticity.

**Figure 6:**
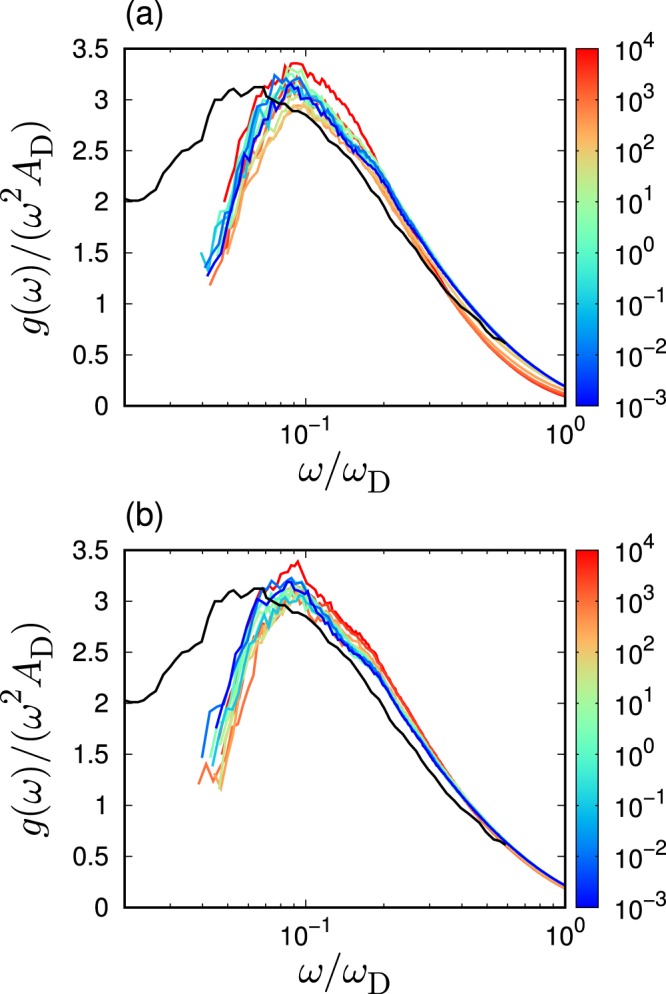
Scaled vibrational spectra. We present the data presented in Fig. 4, in the scaled form: we scale the reduced vDOS $g(\omega )/{\omega }^{2}$ and the frequency $\omega$ by the Debye level ${A}_{{\rm{D}}}$ and the Debye frequency ${\omega }_{{\rm{D}}}$. Here the values of ${A}_{{\rm{D}}}$ and ${\omega }_{{\rm{D}}}$ are presented in Fig. 5. The chain length is (**a**) $L=50$ and (**b**) $L=3$. The color of line indicates the value of bending rigidity ${\varepsilon }_{{\rm{bend}}}$ according to the color bar. Black lines present value of the LJ glass which is taken from ref. ^70^.

To confirm this hypothesis, the scaled vDOS $g(\omega )/({\omega }^{2}{A}_{{\rm{D}}})$ is plotted as a function of the scaled frequency $\omega /{\omega }_{{\rm{D}}}$ and presented in Fig. 6. As discussed above, ${A}_{{\rm{D}}}$ and ${\omega }_{{\rm{D}}}$ are determined mostly by the shear modulus $G$. Although deviations are observed, the scaled vDOSs collapse for different values of ${\varepsilon }_{{\rm{bend}}}$. This result indicates that the effects engendered by the bending rigidity on the low-frequency spectra are comprised of the the shear modulus changes. A same collapse was observed in effects of pressure on the BP in the covalent-bonding network glass (Na${}_{2}$FeSi${}_{3}$O${}_{8}$)^6^. In addition, a previous experiment^20^ demonstrated that the effects of the polymerization are also comprised by the macroscopic elasticity changes. The collapsed results for (a) $L=50$ and (b) $L=3$ are consistent with the experimental observation.

The collapses observed in Fig. 6 indicates that ${\omega }_{{\rm{BP}}}/{\omega }_{{\rm{D}}}$ does not depend on ${\varepsilon }_{{\rm{bend}}}$. As stated previously, when ${\varepsilon }_{{\rm{bend}}}$ varies, ${\omega }_{{\rm{D}}}\propto {\rho }^{1/3}{c}_{{\rm{T}}}\propto {\rho }^{-1/6}\sqrt{G}$. As $\rho$ varies in a range of 15%, as shown in Fig. 1, the effect of $\rho$ on ${\omega }_{{\rm{D}}}$ is weak. Thus, ${\omega }_{{\rm{D}}}$ is approximately proportional to $\sqrt{G}$, which leads to ${\omega }_{{\rm{BP}}}\propto \sqrt{G}$ in the variation of ${\varepsilon }_{{\rm{bend}}}$. The ${\varepsilon }_{{\rm{bend}}}$ dependence of the BP frequency is determined by the shear modulus, which is a macroscopic quantity describing the entire system in an averaged manner. According to the heterogeneous elasticity theory^24–26^, the spatial fluctuations of the local shear modulus $\delta G$ control nature of the BP. The value of $\delta G$ is quantified by the standard deviation of probability distribution function of the local shear modulus^63^. The collapse of $g(\omega )/({\omega }^{2}{A}_{{\rm{D}}})$ as a function of $\omega /{\omega }_{{\rm{D}}}$ indicates that the shear modulus fluctuations relative to the macroscopic value, $\delta G/G$, are constant for all the cases of different bending rigidities. Therefore, the results of this study can be explained as follows. The increase in bending rigidity does not affect the shear modulus fluctuations (relative to the macroscopic moduli) but only affects the macroscopic shear modulus, which leads to the collapse of the scaled vDOS. Furthermore, it is also noted that the recent theoretical study explains the origin of BP in both crystals and glasses in terms of the competition between elastic phonon propagation and diffusive damping^33^. The model based on the phonon Green's function leads to the result ${\omega }_{{\rm{BP}}}\propto \sqrt{G}$. This necessitates further investigations regarding the effects of ${\varepsilon }_{{\rm{bend}}}$ on phonon transport and the phonon's Green function.

#### Participation ratio

To further study the vibrational eigenstates, the participation ratio ${P}^{k}$ that measures the extent of localization of the eigenmodes $k$ is calculated as follows^34,35^:

1 $${P}^{k}=\frac{1}{{N}_{{\rm{p}}}}{\left[\mathop{\sum }\limits_{i=1}^{{N}_{{\rm{p}}}}{({{\boldsymbol{e}}}_{i}^{k}\cdot {{\boldsymbol{e}}}_{i}^{k})}^{2}\right]}^{-1},$$

where $${{\boldsymbol{e}}}_{i}^{k}$$$$(i=1,2,\cdots ,{N}_{{\rm{p}}})$$ are the eigenvectors associated with the eigenfrequencies $${\omega }^{k}$$ ($$i$$ is the index of the monomer particle and $${N}_{{\rm{p}}}$$ is the number of monomer particles). The $${{\boldsymbol{e}}}_{i}^{k}$$ represents the displacements of each monomer bead $$i$$ in the eigenmode $$k$$. It is noted that $${{\boldsymbol{e}}}_{i}^{k}$$ is obtained from the diagonalization of the Hessian matrix and is orthonormalized as $${\sum }_{i=1}^{{N}_{{\rm{p}}}}{{\boldsymbol{e}}}_{i}^{k}\cdot {{\boldsymbol{e}}}_{i}^{l}={\delta }_{kl}$$ ($${\delta }_{kl}$$ is the Kronecker delta). The following extreme cases can occur: $${P}^{k}=2/3$$ for an ideal sinusoidal plane wave, $${P}^{k}=1$$ for an ideal mode in which all constituent particles vibrate equally, and $${P}^{k}=1/{N}_{{\rm{p}}}\ll 1$$ for a perfect localization, which indicates that each vibrational state is associated only with a single atom and that $${e}_{i}^{k}\cdot {e}_{i}^{k}=1$$ for a single $$i$$, otherwise $${e}_{i}^{k}\cdot {e}_{i}^{k}=0$$.

**Figure 7:**
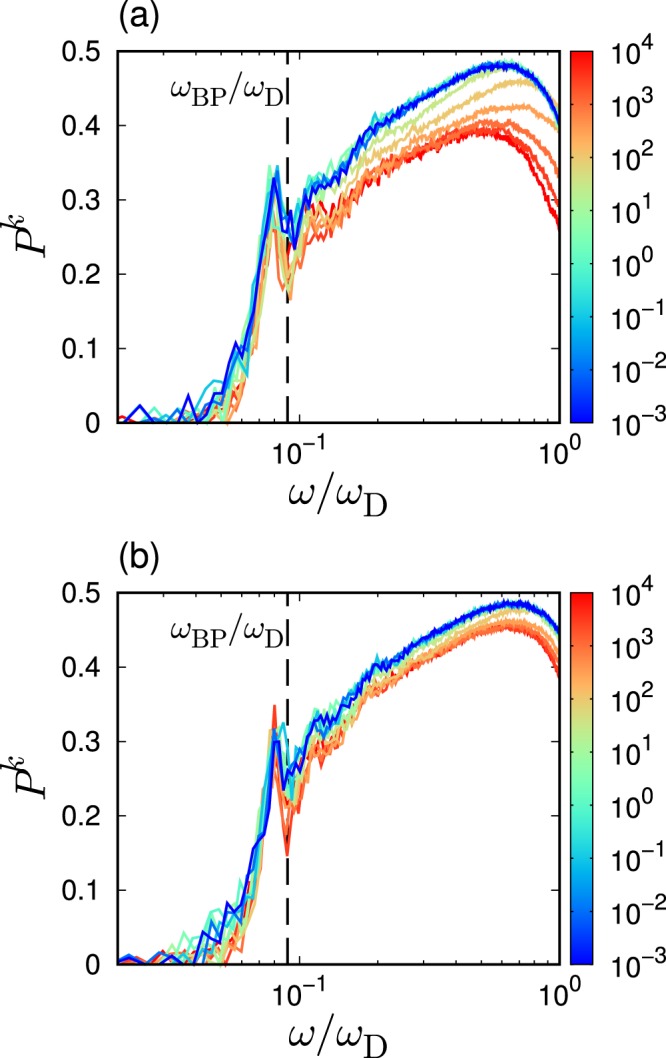
Localization nature of vibrational states. Plots of participation ratio ${P}^{k}$ as a function of the scaled frequency $\omega /{\omega }_{{\rm{D}}}$, for several different bending rigidities of ${\varepsilon }_{{\rm{bend}}}$. The chain length is (**a**) $L=50$ and (**b**) $L=3$. The color of line indicates the value of bending rigidity ${\varepsilon }_{{\rm{bend}}}$ according to the color bar. Data are shown as the average values over bins in the frequency domain of $\left[\omega -\Delta \omega /2,\omega +\Delta \omega /2\right]$ with $\Delta \omega \simeq 0.06$. The vertical line indicates the position of ${\omega }_{{\rm{BP}}}/{\omega }_{{\rm{D}}}$ averaged over the examined systems with varied ${\varepsilon }_{{\rm{bend}}}$.

Figure 7 presents the value of ${P}^{k}$ as a function of the scaled frequency $\omega /{\omega }_{{\rm{D}}}$, for different ${\varepsilon }_{{\rm{bend}}}$. It is noted that the presented data are the binned average values. Below the BP frequency ${\omega }_{{\rm{BP}}}$, ${P}^{k}$ progressively decreases when $\omega$ decreases due to the spatially localized vibrations. The low-frequency localization below ${\omega }_{{\rm{BP}}}$ has also been observed in multiple glasses^34,35,48,49^. Importantly, ${P}^{k}$ below ${\omega }_{{\rm{BP}}}$ collapses between different values of ${\varepsilon }_{{\rm{bend}}}$. This result indicates that the variations of not only the vDOS and the vibrational states due to ${\varepsilon }_{{\rm{bend}}}$ can be characterized by the macroscopic shear modulus changes. However, ${P}^{k}$ does not collapse above ${\omega }_{{\rm{BP}}}$, as also shown in Fig. 7. This result is attributed to the fact that the high-frequency modes above ${\omega }_{{\rm{BP}}}$ reflect microscopic vibrations that cannot be captured by the macroscopic elasticity.

#### Comparison with LJ glasses

The low-frequency spectra are comparable to that of atomic LJ glasses reported in ref. ^70^. As observed in Fig. 4, the height of $g({\omega }_{{\rm{BP}}})/{\omega }_{{\rm{BP}}}^{2}$ of LJ glasses is higher than that of polymer glasses, and ${\omega }_{{\rm{BP}}}$ is lower than that of polymer glasses. These observations are different from the study reported in ref. ^62^, which demonstrated that the low-frequency spectra of polymer glasses correspond to those atomic LJ glasses. In ref. ^62^, the bonded monomers interact via a harmonic potential with a large bonding energy scale of $k=2500$. This value is two orders of magnitude larger than ${\varepsilon }_{{\rm{FENE}}}=30$, investigated in this study. With respect to the large bonding energy, the rigidity of the polymeric chains has a smaller effect on the low-frequency spectra. Therefore, the low-frequency spectra are mainly determined by the non-bonding LJ interactions, whereas the elasticity is mainly determined mainly by the bonding rigidity. As a results, the BP decouples with the macroscopic elasticity, as demonstrated in the previous study^62^.

In contrast to the the results presented in ref. ^62^, the rigidity of the polymeric chains is necessary to determine the elasticity and the low-frequency spectra with respect to the bonding energy scale of ${\varepsilon }_{{\rm{FENE}}}=30$. In fact, the ${\varepsilon }_{{\rm{bend}}}$ reduces the height of $g({\omega }_{{\rm{BP}}})/{\omega }_{{\rm{BP}}}^{2}$, as shown in Fig 4. In this case, the BP couples with the macroscopic elasticity. However, the plot of the scaled $g(\omega )/({\omega }^{2}{A}_{{\rm{D}}})$ as a function of $\omega /{\omega }_{{\rm{D}}}$ does not collapse between the polymer glasses and LJ glass, as shown in Fig. 6. The height of $g(\omega )/({\omega }^{2}{A}_{{\rm{D}}})$ is consistent between the polymer glasses and LJ glass, but $\omega /{\omega }_{{\rm{D}}}$ of the LJ glass is lower than that of the polymer glasses. This result indicates that vibrational states differences between polymer glasses and LJ glasses cannot be described only by changes in macroscopic elasticity, changes in the local elastic properties should be considered as well^6,18,19,38^.

Here we make a note on the finite system size effects. We consider that the present system size of ${N}_{p}\simeq 5000$ is not enough large to sample the low frequency modes below the BP. The previous work on the LJ glass^70^ employed the very large system size up to $N=1000000$ to study the low frequency regime without any system size effect. In contrast, our results of polymer glass for the region below the BP are affected by finite system size effects. However, the system size of ${N}_{p}\simeq 5000$ is enough large to study the vibrational modes in the BP. Indeed, we confirm that our results in the BP regime are not contaminated by the size effects. Thus, as long as we discuss on the BP, we do not need to care for the system size effects.

In addition, the length scale of collective vibrational modes in the BP region is discussed. For atomic LJ glasses, the length scale was evaluated as ${\xi }_{{\rm{BP}}}=2\pi {c}_{{\rm{T}}}/{\omega }_{{\rm{BP}}}$, which corresponds to the size of approximately 23 particle^68^. This length scale diverges near the isostatic point or the marginally stable point, theoretically^27–30^ as well as numerically^41,46,47,73,74^. The present study evaluates the length scale of collective vibrational modes in polymeric glasses as ${\xi }_{{\rm{BP}}}=2\pi {c}_{{\rm{T}}}/{\omega }_{{\rm{BP}}}\approx 12$, which corresponds to half of that for LJ glasses. The vibrational modes in the BP region are more localized nature due to the polymerization. Moreover, the value of ${\xi }_{{\rm{BP}}}$ is independent of the bending rigidity ${\varepsilon }_{{\rm{bend}}}$ because of ${\omega }_{{\rm{BP}}}\propto {\omega }_{{\rm{D}}}\propto {c}_{{\rm{T}}}$. In other words, the bending rigidity does not affect the length scale of the collective vibrational motions in the BP region.

## Discussion

The glass transition temperature, elastic properties, and the low-frequency vibrational spectra were studied in polymeric glasses. In particular, the bending energy scale was highly varied for long chains ($L=50$) and short chains ($L=3$). As the system becomes rigid by increasing the bending rigidity, the glass transition occurs at a higher temperature, leading to a lower density in the glass phase. The lowering density directly affects the isotropic bulk deformation, but does not affect the shear elasticity. The shear elasticity is mainly controlled by the bending rigidity. The non-affinity of polymeric glasses is much larger than that of atomic LJ glasses. This is due to the more complex conformational relaxations of the polymeric chains during non-affine deformation. Even under an isotropic elastic deformation, the non-affine relaxation process should be considered to describe the elastic response.

In addition, it is demonstrated that the BP frequency *ω*_BP_ and its intensity are simply scaled by the Debye frequency *ω*_D_ and the Debye level *A*_D_ which are mainly determined by the macroscopic shear modulus *G*. This result indicates that the BP is controlled by macroscopic shear modulus and that the bending rigidity has a small impact on heterogeneities of local elasticity properties. The effects of the bending rigidity on the BP is similar to that of the polymerization, which has also been explained by macroscopic elasticity changes^20^.

The presented results provide a simple relationship between the BP and the elasticity as well as the glass transition temperature. As the system becomes more rigid by increasing the bending rigidity, the glass transition temperature *T*_g_ and the shear modulus *G* are increased. On the contrary, the bulk modulus $K$ decreases due to the decrease in the density $\rho$ caused by the increase in the glass transition temperature ${T}_{g}$. However, the BP is mainly determined by the shear modulus $G$: ${\omega }_{{\rm{BP}}}\propto {\omega }_{{\rm{D}}}\propto \sqrt{G}$. Therefore, the glass transition temperature, the shear elasticity, and the BP frequency are positively correlated. A similar relationship between ${T}_{g}$ and ${\omega }_{{\rm{BP}}}$ was observed experimentally in ionic liquids systems^75^ and also numerically in LJ glasses^76^. It is noted that the studies of refs. ^75,76^ provided the relationship of ${T}_{g}\propto {\omega }_{{\rm{BP}}}^{2}$, but a clear power-law like relationship between ${T}_{g}$ and ${\omega }_{{\rm{BP}}}$ was not observed in polymeric glasses.

Finally, it is worthwhile to discuss the relationship between structural relaxation above the glass transition temperature and the elastic properties. In fact, there have been proposed the shoving model, which characterizes the activation energy of the structural relaxation time ${\tau }_{\alpha }$ in terms of the shear modulus $G$^77,78^. In the literature, a recent study^79^ has demonstrated the scaling relationship between the structural relaxation time ${\tau }_{\alpha }$ and the Debye-Waller factor $\langle {u}^{2}\rangle$ as ${\tau }_{\alpha }\propto \exp (a{\langle {u}^{2}\rangle }^{-1}+b{\langle {u}^{2}\rangle }^{-2})$ (where $a,b$ are constants) for multiple glass-forming liquids including polymeric glasses. Here, the Debye-Waller factor in the harmonic approximation^80^ is estimated as $\langle {u}^{2}\rangle =3T{\int }_{0}^{\infty }g(\omega )/{\omega }^{2}d\omega \propto T{\omega }_{{\rm{BP}}}^{-2}\propto T{G}^{-1}$. It is naturally expected that the relaxation dynamics become drastically slow by increasing the bending rigidity because of the following relationship:

2 $${\tau }_{\alpha }\propto \exp \left(\alpha \frac{{\omega }_{{\rm{BP}}}^{2}}{T}+\beta \frac{{\omega }_{{\rm{BP}}}^{4}}{{T}^{2}}\right)\propto \exp \left(\alpha ^{\prime} \frac{G}{T}+{\beta }^{^{\prime} }\frac{{G}^{2}}{{T}^{2}}\right),$$

where $\alpha ,\beta ,\alpha ^{\prime} ,{\beta }^{^{\prime} }$ are constants. This simple relationship demonstrates that the BP below ${T}_{g}$ and the structural relaxation above ${T}_{g}$ are well correlated in the polymeric glasses with varying the bending rigidity. Further work is necessary to evaluate its validity by calculating ${\tau }_{\alpha }$, despite the equation analogous to Eq. (2) has empirically been proposed from MD simulations of polymeric glasses^81^.

## Methods

Coarse-grained MD simulations are performed by using the Kremer–Grest model^82^, which treats polymer chains as linear series of monomer beads (particles) of mass $m$. Each polymer chain is composed of $L$ monomer beads, and two cases are considered in this study: long chain length with $L=50$ and short chain length with $L=3$. In a three-dimensional cubic simulation box under periodic boundary conditions, ${N}_{{\rm{p}}}=5000$ and 4998 is defined as the total number of monomers for $L=50$ and $L=3$ respectively, which means that the number of polymeric chains is ${N}_{{\rm{p}}}/L=100$ for $L=50$ and 1666 for $L=3$.

The polymer chain is modeled by three types of inter-particle potentials as follows. Firstly, all the monomer particles interact via the LJ potential:

3 $${U}_{{\rm{LJ}}}(r)=4{\varepsilon }_{{\rm{LJ}}}\,\left[{\left(\frac{\sigma }{r}\right)}^{12}-{\left(\frac{\sigma }{r}\right)}^{6}\right],$$

where $r$ is the distance between two monomers, $\sigma$ is the diameter of monomer, and ${\varepsilon }_{{\rm{LJ}}}$ is the energy scale of the LJ potential. The LJ potential is truncated at the cut-off distance of ${r}_{c}=2.5\sigma$, where the potential and the force (first derivative of the potential) are shifted to zero continuously^70^. Throughout this study, the mass, length, and energy scales are measured in units of $m$, $\sigma$, ${\varepsilon }_{{\rm{LJ}}}$, respectively. The temperature is measured by ${\varepsilon }_{{\rm{LJ}}}/{k}_{{\rm{B}}}$ (${k}_{{\rm{B}}}$ is the Boltzmann constant). Secondly, sequential monomer-beads along the polymeric chain are connected by the FENE potential:

4 $${U}_{{\rm{FENE}}}(r)=\left\{\begin{array}{ll}-\frac{{\varepsilon }_{{\rm{FENE}}}}{2}{R}_{0}^{2}{\rm{ln}}\left[1-{\left(\frac{r}{{R}_{0}}\right)}^{2}\right] & (r\le {R}_{0}),\\ \infty  & (r > {R}_{0}),\end{array}\right.$$

where ${\varepsilon }_{{\rm{FENE}}}$ is the energy scale of the FENE potential, and ${R}_{0}$ is the maximum length of the FENE bond. Their values are defined as ${\varepsilon }_{{\rm{FENE}}}=30$ and ${R}_{0}=1.5$, according to ref. ^61^. Finally, three consecutive monomer beads along the chain interact via the bending potential defined as follows:

5 $${U}_{{\rm{bend}}}(\theta )={\varepsilon }_{{\rm{bend}}}\left[1-\cos (\theta -{\theta }_{0})\right],$$

where $\theta$ is the angle formed by three consecutive beads, and ${\varepsilon }_{{\rm{bend}}}$ is the associated energy scale. This potential intends to stabilize the angle $\theta$ at ${\theta }_{0}$ that we set as ${\theta }_{0}=109.{5}^{\circ }$. Here, the value of ${\varepsilon }_{{\rm{bend}}}$ in a wide range from ${\varepsilon }_{{\rm{bend}}}=1{0}^{-3}$ to $1{0}^{4}$, and the effects of the bending rigidity on the vibrational and mechanical properties of the polymeric system are studied.

MD simulations are performed by using the Large-scale Atomic/Molecular Massively Parallel Simulator (LAMMPS)^83^. The polymeric system is first equilibrated in the melted, liquid state at a temperature $T=1.0$. Further, the system is cooled down under a fixed pressure condition of $p=0$ and with a cooling rate of $dT/dt=1{0}^{-4}$. During the cooling process, the glass transition occurs at a particular temperature, i.e., the glass transition temperature. After the glass transition, the system is quenched down towards the zero temperature, i.e., $T=0$ state.

## Supplementary information

Supplementary Information

## Data Availability

The data supporting the findings of this study are available from the corresponding authors upon reasonable request.
