# Supplementary material for: Boson peak, elasticity, and glass transition temperature in polymer glasses: Effects of the rigidity of chain bending
Source: Sci Rep. 2019 Dec 20;9:19514. doi: 10.1038/s41598-019-55564-2 (PMC6925306; doi:10.1038/s41598-019-55564-2)
Supplement: Supplementary file 1 — Supplementary Information [file 41598_2019_55564_MOESM1_ESM.pdf]

Supplementary Material

Boson peak, elasticity, and glass transition temperature in polymer glasses: Effects of the rigidity of chain bending

Naoya Tomoshige, Hideyuki Mizuno, Tatsuya Mori, Kang Kim, and Nobuyuki Matubayasi

S.1. FORMALISM OF THE HESSIAN MATRIX

The Hessian matrix of the interaction potential $U(\boldsymbol{r})$ is generally expressed as follows:

$$H_{nm}^{ab} = \frac{\partial^2 U(\boldsymbol{r})}{\partial r_n^a \partial r_m^b} \quad (a, b = x, y, x) \tag{S.1}$$

where n and m denote the particle number index (n, m=1, 2, $\cdots$, N_p). As given in Ref. [S1], the following expressions are useful using a generic argument z for the first and second derivatives of $U(z)$:

$$\frac{\partial U(z)}{\partial x} = \frac{\partial U(z)}{\partial z}\frac{\partial z}{\partial x}, \tag{S.2}$$

$$\frac{\partial U^2(z)}{\partial x \partial y} = \frac{\partial U^2(z)}{\partial^2 z}\frac{\partial z}{\partial x}\frac{\partial z}{\partial y} + \frac{\partial U(z)}{\partial z}\frac{\partial^2 z}{\partial x \partial y} = c\frac{\partial z}{\partial x}\frac{\partial z}{\partial y} + t\frac{\partial^2 z}{\partial x \partial y}. \tag{S.3}$$

S.1 (a). Two-body interaction

For two-body interactions (FENE and LJ potentials), the distance between particles i and j, $z = |\boldsymbol{r}_j - \boldsymbol{r}_i| = r_{ij}$ is used and the following relationships are obtained:

$$H_{nm}^{ab} = \frac{\partial^2 U(r_{ij})}{\partial r_n^a \partial r_m^b} = c_{ij}\frac{\partial r_{ij}}{\partial r_n^a}\frac{\partial r_{ij}}{\partial r_m^b} + t_{ij}\frac{\partial^2 r_{ij}}{\partial r_n^a \partial r_m^b}, \tag{S.4}$$

with

$$c_{ij} = \frac{\partial^2 U(r_{ij})}{\partial r_{ij}^2}, \quad t_{ij} = \frac{\partial U(r_{ij})}{\partial r_{ij}}, \tag{S.5}$$

and

$$\frac{\partial r_{ij}}{\partial r_n^a} = (\delta_{nj} - \delta_{ni})\,\hat{n}_{ij}^a, \tag{S.6}$$

$$\frac{\partial^2 r_{ij}}{\partial r_n^a \partial r_m^b} = \frac{1}{r_{ij}}(\delta_{nj} - \delta_{ni})(\delta_{mj} - \delta_{mi})\left(\delta_{ab} - \hat{n}_{ij}^a \hat{n}_{ij}^b\right), \tag{S.7}$$

where, $\hat{n}_{ij} = \boldsymbol{r}_{ij}/r_{ij}$ is the unit vector between the particles i and j. These expressions are same as those presented in Ref. [S1].

S.1 (b). Three-body interaction

For three-body interactions (bending potential), the bond angle of particles i, j, and k is used as follows:

$$z = \theta_{ijk} = \arccos\frac{(\boldsymbol{r}_j - \boldsymbol{r}_i)\cdot(\boldsymbol{r}_k - \boldsymbol{r}_i)}{r_{ij}r_{ki}} = \arccos A_{ijk}, \tag{S.8}$$

hence,

$$H_{nm}^{ab} = \frac{\partial^2 U\left(\theta_{ijk}\right)}{\partial r_n^a \partial r_m^b} = \tilde{c}_{ijk} \frac{\partial \theta_{ijk}}{\partial r_n^a} \frac{\partial \theta_{ijk}}{\partial r_m^b} + \tilde{t}_{ijk} \frac{\partial^2 \theta_{ijk}}{\partial r_n^a \partial r_m^b} \tag{S.9}$$

with

$$\tilde{c}_{ijk} = \frac{\partial^2 U\left(\theta_{ijk}\right)}{\partial \theta_{ijk}^2}, \quad \tilde{t}_{ijk} = \frac{\partial U\left(\theta_{ijk}\right)}{\partial \theta_{ijk}}. \tag{S.10}$$

This following expression is obtained:

$$H_{nm}^{ab} = \frac{\tilde{c}_{ijk}}{\sin^2 \theta_{ijk}} \frac{\partial A_{ijk}}{\partial r_n^a} \frac{\partial A_{ijk}}{\partial r_m^b} - \frac{\tilde{t}_{ijk}}{\sin \theta_{ijk}} \left[\frac{\cos \theta_{ijk}}{\sin^2 \theta_{ijk}} \frac{\partial A_{ijk}}{\partial r_n^a} \frac{\partial A_{ijk}}{\partial r_m^b} + \frac{\partial^2 A_{ijk}}{\partial r_n^a \partial r_m^b} \right], \tag{S.11}$$

with

$$\frac{\partial A_{ijk}}{\partial r_n^a} = \frac{1}{r_{ij}} \left(\delta_{nj} - \delta_{ni}\right) \left(\hat{n}_{ik}^a - \hat{n}_{ij}^a \cos \theta_{ijk}\right) + \frac{1}{r_{ik}} \left(\delta_{nk} - \delta_{ni}\right) \left(\hat{n}_{ij}^a - \hat{n}_{ik}^a \cos \theta_{ijk}\right),$$

$$\begin{aligned} \frac{\partial^2 A_{ijk}}{\partial r_n^a \partial r_m^b} = & \frac{\delta_{ji}^n \delta_{ji}^m}{r_{ij}^2} \left[\left(3 \hat{n}_{ij}^a \hat{n}_{ij}^b - \delta_{ab}\right) \cos \theta_{ijk} - \left(\hat{n}_{ik}^a \hat{n}_{ij}^b + \hat{n}_{ij}^a \hat{n}_{ik}^b\right) \right] \\ & + \frac{\delta_{ji}^n \delta_{ki}^m}{r_{ij} r_{ik}} \left[\delta_{ab} + \hat{n}_{ij}^a \hat{n}_{ik}^b \cos \theta_{ijk} - \left(\hat{n}_{ik}^a \hat{n}_{ik}^b + \hat{n}_{ij}^a \hat{n}_{ij}^b\right) \right] \\ & + \frac{\delta_{ki}^n \delta_{ji}^m}{r_{ij} r_{ik}} \left[\delta_{ab} + \hat{n}_{ik}^a \hat{n}_{ij}^b \cos \theta_{ijk} - \left(\hat{n}_{ik}^a \hat{n}_{ik}^b + \hat{n}_{ij}^a \hat{n}_{ij}^b\right) \right] \\ & + \frac{\delta_{ki}^n \delta_{ki}^m}{r_{ik}^2} \left[\left(3 \hat{n}_{ik}^a \hat{n}_{ik}^b - \delta_{ab}\right) \cos \theta_{ijk} - \left(\hat{n}_{ij}^a \hat{n}_{ik}^b + \hat{n}_{ik}^a \hat{n}_{ij}^b\right) \right]. \end{aligned} \tag{S.12}$$

The differences between the proposed calculation and the expression defined in Ref. [S1] arise from Eq. (S.7) and the second term in the r.h.s. of Eq. (S.11). The overall profile of the vDOS $g(\omega)$ is not affected by implementing the diagonalization of the Hessian matrix using the expressions in Ref. [S1]. A certain number of negative frequency eigenmodes that have been reported in Ref. [S1] have also been observed. On the contrary, the presented results of $g(\omega)$ using Eqs. (S.7) and (S.11) do not exhibit any negative eigenfrequency modes (see Fig. 4 in the main text).

[S1] R. Milkus, C. Ness, V. V. Palyulin, J. Weber, A. Lapkin, and A. Zaccone, Macromolecules **51**, 1559 (2018).
